# Tobacco Advertising During the COVID-19 Pandemic in Japan

**DOI:** 10.2188/jea.JE20210151

**Published:** 2021-07-05

**Authors:** Masao Ichikawa, Haruhiko Inada, Ai Hori, Takahiro Tabuchi

**Affiliations:** 1 Department of Global Public Health, Faculty of Medicine, University of Tsukuba, Tsukuba, Ibaraki, Japan; 2 Department of International Health, Johns Hopkins Bloomberg School of Public Health, Baltimore, Maryland, USA; 3 Cancer Control Center, Osaka International Cancer Institute, Osaka, Japan

**Keywords:** tobacco industry, COVID-19, advertisement, media

A recent study published in this journal reported that tobacco consumption had increased among smokers during the pandemic, especially among those in younger age groups, working from home or living alone.^1^ While their living circumstances might have prompted them to smoke more, their smoking behaviors could have been influenced by tobacco advertisements.^2^ We, therefore, investigated the tobacco-related ad volume in newspapers and magazines before and during the pandemic.

We obtained the data of tobacco advertisements from MRS Advertising Research, Inc., which compiles various industries’ advertisements in newspapers and magazines. The data included the number of newspapers and magazines from which advertisements were extracted, the ad volume related to tobacco products, and all kinds of advertisements in newspapers and magazines published from 2011 to 2020. The ad volume was calculated based on the size of ads, and the unit of the ad volume was 1/15 page for newspapers and one page for magazines. Thus, full-page advertisements in newspapers were counted as 15, while those on half a page in magazines were counted as 0.5. Ads such as event announcements and manner ads were excluded.

Table 1 shows the number of newspapers and magazines from which ads were extracted, the annual volume of tobacco ads and all kinds of ads, and the proportion of tobacco ads to all advertisements in newspapers and magazines between 2011 and 2020. The number of newspapers and magazines slightly differed during this period, ranging from 119 to 121 for newspapers and from 355 to 375 for magazines. The volume of tobacco ads in newspapers was highest in 2020. Accordingly, the proportion of tobacco ads to all ads in 2020 was the highest of the past decade. Meanwhile, the volume of tobacco ads in magazines in 2020 was the second-lowest of the past decade, possibly due to the smaller number of magazines, which was 355 in 2020, compared with the average of 369 in other years. The proportion of tobacco ads to all ads in 2020 in magazines was, however, highest in the past decade.

**Table 1.** Number of newspapers and magazines from which ads were extracted, annual volume of tobacco ads and all ads,^a^ and proportion of tobacco ads to all ads in newspapers and magazines from 2011 to 2020

Year	Newspapers				Magazines			
	
	Number	Tobacco ads	All ads	%	Number	Tobacco ads	All ads	%
2011	119	1,979	4,792,732	0.04%	367	1,312	188,994	0.69%
2012	119	2,342	5,046,739	0.05%	370	1,857	191,008	0.97%
2013	120	710	5,202,422	0.01%	373	1,642	186,679	0.88%
2014	120	3,755	5,221,343	0.07%	375	1,848	176,048	1.05%
2015	121	2,452	5,125,202	0.05%	373	1,730	165,424	1.05%
2016	120	1,324	5,020,937	0.03%	371	1,317	152,132	0.87%
2017	120	2,730	4,933,338	0.06%	371	1,836	141,916	1.29%
2018	120	4,104	4,734,465	0.09%	364	1,209	129,635	0.93%
2019	120	3,621	4,582,691	0.08%	361	1,404	117,040	1.20%
2020	120	5,485	4,285,149	0.13%	355	1,279	93,110	1.37%

^a^The unit of ad volume is 1/15 page for newspapers and one page for magazines. Therefore, full-page ads in newspapers were counted as 15, while ads on half a page in magazines were counted as 0.5.

The tobacco industry continued to promote its products during the COVID-19 pandemic, with the highest ad volume in newspapers and the highest proportion of tobacco ads to all ads in both newspapers and magazines in the past decade. Thus far, no lockdown has been implemented in Japan to control the pandemic, but working and eating at home has been advocated and rapidly adopted as part of a ‘new normal’ lifestyle.^3^ This lifestyle change might have provided an opportunity for the tobacco industry to promote its products as smoking has been prohibited in most public spaces, including offices and restaurants, but not at home.

It should be noted that we did not determine whether continued tobacco ads influenced tobacco consumption during the pandemic because this was beyond the aim of our investigation. Moreover, we did not examine advertisements on television and the Internet, though major national and local newspapers and magazines (nearly 500) were covered in the analysis. In sum, tobacco ads persisted in newspapers and magazines during the pandemic, when people were encouraged to stay at home where smoking was not prohibited, unlike in offices or restaurants.
